# A boundary value approach for solving three-dimensional elliptic and hyperbolic partial differential equations

**DOI:** 10.1186/s40064-015-1348-1

**Published:** 2015-10-09

**Authors:** T. A. Biala, S. N. Jator

**Affiliations:** Department of Mathematics and Computer Science, Jigawa State University, Kafin Hausa, P.M.B 048, Kafin Hausa, Nigeria; Department of Mathematics and Statistics, Austin Peay State University, Clarksville, TN 37044 USA

**Keywords:** Boundary value methods, Method of lines, Hyperbolic and elliptic PDEs, Systems of ordinary differential equations, 65N06, 65N40

## Abstract

In this article, the boundary value method is applied to solve three dimensional elliptic and hyperbolic partial differential equations. The partial derivatives with respect to two of the spatial variables (*y*, *z*) are discretized using finite difference approximations to obtain a large system of ordinary differential equations (ODEs) in the third spatial variable (*x*). Using interpolation and collocation techniques, a continuous scheme is developed and used to obtain discrete methods which are applied via the Block unification approach to obtain approximations to the resulting large system of ODEs. Several test problems are investigated to elucidate the solution process.

## Background

This paper is devoted to the numerical computation of the three dimensional elliptic and hyperbolic PDEs. The general three dimensional problem (without cross derivatives) is given as

1 $$\begin{aligned} a\dfrac{\partial ^2 u}{\partial x^2} + b\dfrac{\partial ^2 u}{\partial y^2} + c\dfrac{\partial ^2 u}{\partial z^2} + d\dfrac{\partial u}{\partial x} + e\dfrac{\partial u}{\partial y} + f\dfrac{\partial u}{\partial z} = G(x,y,z,u),\quad (x,y,z) \in \Omega \subset \mathbb {R}^3 \end{aligned}$$

defined in the domian $\Omega = \{(x,y,z)/(x,y,z) \in (L_1, L_2, L_3) \times (L_4, L_5, L_6)\}$ with boundary $\partial \Omega$. Associated with (1) are the initial conditions

$$\begin{aligned} u(L_1, y, z) = g_1(y, z), \quad u_x(L_1,y,z) = g_2(y, z) \qquad \quad (y,z) \in (L_2, L_3) \times (L_5, L_6) \end{aligned}$$

or the boundary conditions

$$\begin{aligned} u(L_1, y, z) = g_1(y, z), \quad u(L_4,y,z) = g_3(y, z) \qquad \quad (y,z) \in (L_2, L_3) \times (L_5, L_6) \end{aligned}$$

and the Dirichlet boundary conditions

$$\begin{aligned} u(x, L_2, z) = h_1(x, z), &{} \qquad \quad u(x,L_5,z) = h_2(x, z)\\ u(x, y, L_3) = h_3(x, y), &{} \qquad \quad u(x,y,L_6) = h_4(x, y)\\ \end{aligned}$$

The unknown function *u*, the variable coefficients *a*, *b*, *c*, *d*, *e*, *f* and the forcing term G are assumed to be continuously differentiable and have the required partial derivatives on $\Omega$.

The numerical approximation of (1) has received great attention from researchers in the past few decades. This is because most modeled physical and engineering processes results in PDEs of the form (1). The standard convection diffusion equation is obtained if $a=b=c\ne 0$ and $G\equiv G(x,y,z)$ for all $x, y, z \in \Omega$ (see Ge and Zhang 2002). Many transport processes including fluid flows and heat transfer can be modeled by a convective diffusive equation which describes the convection and diffusion of various physical quantities such as heat and momentum (see Roache 1976). Several numerical schemes have been developed for (1) which include the compact difference scheme (Ge and Zhang 2002; Spotz 1995; Spotz and Carey 1996; Zhang 1998a, b, c, d; Zhang et al. 2000), compact alternating direct implicit scheme (Cui 2010; Liao and Sun 2010), finite difference methods (Jain et al. 1992; Mohanty and Singh 2006), method of lines (Brastos 2007; Dehghan and Shokri 2008) , Jacobi elliptic function method (Bhrawy and Abdelkawy 2013), the use of collocation and radial basis functions (Shakeri and Dehghan 2008), Riccati–Bernoulli sub ODE method (Yang et al. 2015), the use of the expansion methods (He 1998; Roshid and Rahman 2014; Alam et al. 2014; Roshid et al. 2013, 2014a, b, c) among others.

 The method of lines approach is commonly used for solving PDEs whereby the PDEs is converted into a system of ODEs by replacing the appropriate derivatives by finite difference approximations. Our objective is to convert the 3D PDEs into a system of ODEs by replacing two of the spatial derivatives by central difference approximations. The resulting system is then solved using the BVM. Specifically, we discretize the *y* and *z* variable with mesh spacings

$$\begin{aligned} \Delta y = \dfrac{L_5 - L_2}{M} \qquad y_m = L_2 + m\Delta y, \quad m=0(1)M \end{aligned}$$

$$\begin{aligned} \Delta z = \dfrac{L_6 - L_3}{N} \qquad z_n = L_3 + n\Delta z, \quad n=0(1)N \end{aligned}$$

We then define the vectors

$$\begin{aligned} \mathbf {u} = [u_{1,1}(x), u_{1,2}(x), u_{2,1}(x), \ldots , u_{m-1, n-1}(x)]^T \end{aligned}$$

and

$$\begin{aligned} \mathbf {G} = [G_{1,1}(x), G_{1,2}(x), G_{2,1}(x), \ldots , G_{m-1, n-1}(x)]^T \end{aligned}$$

where $$u_{m,n}(x) \approx u(x, y_m, z_n)$$ and $$G_{m,n}(x) = \approx G(x,y_m, z_n, u_{m,n})$$.

Also, we replace the partial derivatives $\dfrac{\partial ^2 u(x,y,z)}{\partial y^2}$, $\dfrac{\partial ^2 u(x,y,z)}{\partial z^2}$, $\dfrac{\partial u(x,y,z)}{\partial y}$ and $\dfrac{\partial u(x,y,z)}{\partial z}$ occuring in (1) by central difference approximations

$$\begin{aligned} \dfrac{\partial ^2 u(x,y,z)}{\partial y^2} &{}= \dfrac{u(x, y_{m+1}, z_n) - 2u(x,y_m, z_n) + u(x, y_{m-1}, z_n)}{(\Delta y)^2}\\ \dfrac{\partial ^2 u(x,y,z)}{\partial z^2} &{}= \dfrac{u(x, y_{m}, z_{n+1}) - 2u(x,y_m, z_n) + u(x, y_{m}, z_{n-1})}{(\Delta z)^2}\\ \dfrac{\partial u(x,y,z)}{\partial y} &{}= \dfrac{u(x, y_{m+1}, z_n) - u(x, y_{m-1}, z_n)}{2(\Delta y)}\\ \dfrac{\partial u(x,y,z)}{\partial z} &{}= \dfrac{u(x, y_{m}, z_{n+1}) - u(x, y_{m}, z_{n-1})}{2(\Delta z)}\\ \end{aligned}$$

Problem (1) leads to the semidiscretized system

2 $$\begin{aligned} \dfrac{d^2u_{m,n}}{dx^2} &{}= \dfrac{1}{a_{m,n}}\left\{ -b_{m,n}\left[ \dfrac{u_{m+1,n}-2u_{m,n} + u_{m-1,n}}{(\Delta y)^2}\right] - c_{m,n}\left[ \dfrac{u_{m,n+1}-2u_{m,n} + u_{m,n-1}}{(\Delta z)^2}\right] \right. \\ &{}\left. \quad + \, d_{m,n} \dfrac{du_{m,n}}{dx} + e_{m,n} \dfrac{u_{m+1,n} - u_{m-1,n}}{(2\Delta y)} + f_{m,n} \dfrac{u_{m,n+1} - u_{m,n-1}}{(2\Delta z)} + G_{m,n}\right\} \end{aligned}$$

which can be written in the form

3 $$\begin{aligned} \mathbf {u''} = \mathbf {f}(x,\mathbf {u, u'}) \end{aligned}$$

subject to the initial conditions

4 $$\begin{aligned} \mathbf {u}(L_1) = \mathbf {u}_0 \quad \text {and} \quad \mathbf {u'}(L_1) = \mathbf {u'}_0 \end{aligned}$$

or the boundary conditions

5 $$\begin{aligned} \mathbf {u}(L_1) = \mathbf {u}_0 \quad \text {and} \quad \mathbf {u}(L_4) = \mathbf {u}_{m,n} \end{aligned}$$

where $\mathbf {f}(x,\mathbf {u, u'}) = \mathbf {Au + G}$ and $\mathbf {A}$ is a $k\times k$ matrix $(k = (M-1)(N-1))$ arising from the semidiscretized system (2) which is expressed in the form (3) and whose solution is sought using the BVMs. We note that $\mathbf {G}$ is a vector of constants. The BVMs are a class of linear multistep methods (LMM) with step number *k* and whose *k* additional conditions are not only imposed at the beginning of the integration process but also at the end so that they form a discrete analog of the continuous boundary value problems. Thus, they are used for the numerical approximation of both initial and boundary value problems. They have been used for the solution of first order 1D initial and boundary value problems and their convergence and linear stability properties have been fully discussed in Brugnano and Trigiante (1998). Recently, Biala and Jator (2015) developed BVMs for the direct solution of systems of the general second order ODEs. One main feature of the BVMs is that they can be used in the same way for solving both initial and boundary value problems. Therefore, such methods are the best candidate for solving the semididscretized PDEs in (3). The BVMs simultaneously generates approximate solutions $(u_{1,m,n}, u_{2,m,n}, \ldots , u_{l,m,n})^T$ to the exact solution $(u_{m,n}(x_1), u_{m,n}(x_2), \ldots , u_{m,n}(x_l))^T$ on the entire interval of integration. This approach has the advantage of producing smaller global errors than those produced by the step-by-step methods due to the presence of accumulated errors at each step in the step-by-step method.

The paper is organized as follows: in "Description of the BVM", we derive a continuous scheme which is used to formulate the BVM as well as investigate the properties of the BVM. The computational complexities associated with the method is addressed in "Computational procedures". Several numerical test examples are given in "Numerical examples" to show the accuracy of the method. We give some concluding remarks in "Conclusion".

## Description of the BVM

We propose a BVM for (1) in which on the partition $\pi _L, h>0, x_l = x_0 + lh, l=0,1, \ldots , L,$ the four step $[x_{l},u_{l,m,n},u'_{l,m,n}] \mapsto [x_{l+4},u_{l+4,m,n},u'_{l+4,m,n}]$ is given by the equations

6 $$\begin{aligned} \alpha _{1,0}u_{l,m,n}+\alpha _{1,1}u_{l+1,m,n}+\alpha _{1,2}u_{l+2,m,n}&{}=h^{2}(\beta _{1,0}f_{l,m,n}+\beta _{1,1}f_{l+1,m,n}+\beta _{1,2}f_{l+2,m,n}+\beta _{1,3}f_{l+3,m,n}\\ &{} \quad + \, \beta _{1,4}f_{l+4,m,n}),\\ \alpha _{2,0}u_{l,m,n}+\alpha _{2,2}u_{l+2,m,n}+\alpha _{2,3}u_{l+3,m,n}&{}=h^{2}(\beta _{2,0}f_{l,m,n}+\beta _{2,1}f_{l+1,m,n}+\beta _{2,2}f_{l+2,m,n}+\beta _{2,3}f_{l+3,m,n}\\ &{} \quad + \, \beta _{2,4}f_{l+4,m,n}),\\ \alpha _{3,0}u_{l,m,n}+\alpha _{3,2}u_{l+2,m,n}+\alpha _{3,3}u_{l+4,m,n}&{}=h^{2}(\beta _{3,0}f_{l,m,n}+\beta _{3,1}f_{l+1,m,n}+\beta _{3,2}f_{l+2,m,n}+\beta _{3,3}f_{l+3,m,n}\\ &{} \quad + \, \beta _{3,4}f_{l+4,m,n}),\\ \alpha _{4,0}u_{l,m,n}+\alpha _{4,2}u_{l+2,m,n}+h\alpha '_{4,0}u'_{l,m,n}&{}=h^{2}(\beta _{4,0}f_{l,m,n}+\beta _{4,1}f_{l+1,m,n}+\beta _{4,2}f_{l+2,m,n}+\beta _{4,3}f_{l+3,m,n}\\ &{} \quad + \,\beta _{4,4}f_{l+4,m,n}),\\ \alpha _{5,0}u_{l,m,n}+\alpha _{5,2}u_{l+2,m,n}+h\alpha '_{5,1}u'_{l+1,m,n}&{}=h^{2}(\beta _{5,0}f_{l,m,n}+\beta _{5,1}f_{l+1,m,n}+\beta _{5,2}f_{l+2,m,n}+\beta _{5,3}f_{l+3,m,n}\\ &{} \quad + \, \beta _{5,4}f_{l+4,m,n}),\\ \alpha _{6,0}u_{l,m,n}+\alpha _{6,2}u_{l+2,m,n}+h\alpha '_{6,2}u'_{l+2,m,n}&{}=h^{2}(\beta _{6,0}f_{l,m,n}+\beta _{6,1}f_{l+1,m,n}+\beta _{6,2}f_{l+2,m,n}+\beta _{6,3}f_{l+3,m,n}\\ &{} \quad + \, \beta _{6,4}f_{l+4,m,n}),\\ \alpha _{7,0}u_{l,m,n}+\alpha _{7,2}u_{l+2,m,n}+h\alpha '_{7,3}u'_{l+3,m,n}&{}=h^{2}(\beta _{7,0}f_{l,m,n}+\beta _{7,1}f_{l+1,m,n}+\beta _{7,2}f_{l+2,m,n}+\beta _{7,3}f_{l+3,m,n}\\ &{} \quad + \, \beta _{7,4}f_{l+4,m,n}),\\ \alpha _{8,0}u_{l,m,n}+\alpha _{8,2}u_{l+2,m,n}+h\alpha '_{8,4}u'_{l+4,m,n}&{}=h^{2}(\beta _{8,0}f_{l,m,n}+\beta _{8,1}f_{l+1,m,n}+\beta _{8,2}f_{l+2,m,n}+\beta _{8,3}f_{l+3,m,n}\\ &{} \quad + \,\beta _{8,4}f_{l+4,m,n}). \end{aligned}$$

where $\pi _L: L_1 = x_0 < x_1 < x_2 < \cdots < x_L = L_4$ and $\alpha _{i,j}, \alpha '_{i,j}$, $\beta _{i,j}$, $i=1(1)8$ and $j=0(1)4$ are coefficients to be uniquely determined. We note that $u_{l+i,m,n}$ denote the numerical approximation to the analytical solution $u(x_{l+i},y_m,z_n)$ and $f_{{l+i},m,n} \equiv f(x_{l+i},y_m, z_n)$.

### Development of the continuous BVM

In this section, we discuss the construction of the continuous scheme, via the interpolation and collocation approach (Jator and Li 2012), from which (6) is derived.

#### **Theorem 1**

*Let the continuous representation*

7 $$\begin{aligned} \Phi (x) = \alpha _2(x)u_{l+2,m,n} + \alpha _0(x)u_{l,m,n} + h^2\sum _{j=0}^{4}\beta _j(x)f_{l+j,m,n} \end{aligned}$$

*satisfy the following conditions*

8 $$\begin{aligned} \Phi (x_{l+i}) &{}= u_{l+i,m,n}, \qquad i=0,2\\ \Phi ''(x_{l+j}) &{}= f_{l+j,m,n}, \qquad j=0(1)4 \end{aligned}$$

*then the continuous representation* (7)* is equivalent to*

9 $$\begin{aligned} \Phi (x) = \sum _{j=0}^{6}\dfrac{det(V_j)}{det(V)}P_j(x) \end{aligned}$$

*where we define the matrix V as*

$$\begin{aligned} V =\left( \begin{array}{ccc} P_0(x_{l})&{} \cdots &{} P_6(x_{l}) \\ P_0(x_{l+2})&{} \cdots &{} P_6(x_{l+2}) \\ P''_0(x_{l})&{} \cdots &{} P''_6(x_{l}) \\ P''_0(x_{l+1})&{} \cdots &{} P''_6(x_{l+1}) \\ \vdots &{} \vdots &{} \vdots \\ P''_0(x_{l+4})&{} \cdots &{} P''_6(x_{l+4}) \\ \end{array} \right) , \end{aligned}$$

$V_j$*is obtained by replacing thejth column ofV byW whereTdenotes the transpose,*$P_j(x) = x^j, j= 0(1)6$*are basis functions and W is a vector given by*

$$\begin{aligned} W = (u_{l,m,n}, u_{l+2,m,n}, f_{l,m,n}, f_{l+1,m,n}, \ldots , f_{l+4,m,n})^T. \end{aligned}$$

#### *Proof*

We define the polynomial basis functions

10 $$\begin{aligned} \alpha _j(x) &{}= \sum _{i=0}^{6}\alpha _{i+1,j}P_i(x), \quad j=0,1\\ h^2\beta _j(x) &{}= \sum _{i=0}^{6}\beta _{i+1,j}P_i(x), \quad j=0(1)4 \end{aligned}$$

where $\alpha _{i+1,j}$ and $h^2\beta _{i+1,j}$ are coefficients to be uniquely determined.

Substituting (10) into (7), we have

$$\begin{aligned} \Phi (x) = \sum _{i=0}^{6}\sum _{j=0}^{1}\alpha _{i+1,j}P_i(x)u_{l+j,m,n} + \sum _{i=0}^{6}\sum _{j=0}^{4}h^2\beta _{i+1,j}P_i(x)f_{l+j,m,n} \end{aligned}$$

which may be written as

$$\begin{aligned} \Phi (x) = \sum _{i=0}^{6}\left[ \sum _{j=0}^{1}\alpha _{i+1,j}u_{l+j,m,n} + \sum _{j=0}^{4}h^2\beta _{i+1,j}f_{l+j,m,n}\right] P_i(x) \end{aligned}$$

and expressed in the form

11 $$\begin{aligned} \Phi (x) = \sum _{i=0}^{6}\ell _iP_i(x) \end{aligned}$$

where

$$\begin{aligned} \ell _i = \sum _{j=0}^{1}\alpha _{i+1,j}u_{l+j,m,n} + \sum _{j=0}^{4}h^2\beta _{i+1,j}f_{l+j,m,n} \end{aligned}$$

Imposing conditions (8) on (11), we obtain a system of seven equations, which can be expressed as $V = LW$ where $L=(\ell _0, \ell _1, \ldots , \ell _6)^T$ is a vector of seven undetermined coefficients. Using the Crammer’s rule, the elements of *L* can be obtained and are given as

$$\begin{aligned} \ell _i = \dfrac{det(V_j)}{det(V)}, \quad j=0(1)4 \end{aligned}$$

where $V_j$ is obtained by replacing the *jth* column of *V* by *W*. We rewrite (11) as (9) using the newly found elements of *L*. $\square$

### BVM and its block extension

The coefficients given in (6) are specified by evaluating (9) at $x=\{x_{l+1}, x_{l+3}, x_{l+4}\}$ and evaluating $\Phi '(x)$ at $x=\{x_l, x_{l+1}, x_{l+2}, x_{l+3}, x_{l+4}\}$ to obtain

12 $$\begin{aligned} u_{l+1,m,n} - \frac{1}{2}u_{l+2,m,n} - \frac{1}{2}u_{l,m,n} &{}= \dfrac{h^2}{480}\left( -19f_{l,m,n} - 204f_{l+1,m,n} - 14f_{l+2,m,n}- 4f_{l+3,m,n} + f_{l+4,m,n}\right) \\ u_{l+3,m,n} - \frac{3}{2}u_{l+2,m,n} + \frac{1}{2}u_{l,m,n} &{}= \dfrac{h^2}{480}\left( 17f_{l,m,n} + 252f_{l+1,m,n} + 402f_{l+2,m,n} + 52f_{l+3,m,n} - 3 f_{l+4,m,n}\right) \\ u_{l+4,m,n} - 2u_{l+2,m,n} - u_{l,m,n} &{}= \dfrac{h^2}{15}\left( f_{l,m,n} + 16f_{l+1,m,n} + 26f_{l+2,m,n} + 16f_{l+3,m,n} + 257f_{l+4,m,n}\right) \\ hu'_{l,m,n} - \frac{1}{2}u_{l+2,m,n} + \frac{1}{2}u_{l,m,n} &{}= \dfrac{h^2}{180}\left( -53f_{l,m,n} - 144f_{l+1,m,n} + 30f_{l+2,m,n} - 16f_{l+3,m,n} + 3f_{l+4,m,n}\right) \\ hu'_{l+1,m,n} - \frac{1}{2}u_{l+2,m,n} + \frac{1}{2}u_{l,m,n} &{}= \dfrac{h^2}{720}\left( 39f_{l,m,n} + 70f_{l+1,m,n} - 144f_{l+2,m,n} + 42f_{l+3,m,n} - 7f_{l+4,m,n}\right) \\ hu'_{l+2,m,n} - \frac{1}{2}u_{l+2,m,n} + \frac{1}{2}u_{l,m,n} &{}= \dfrac{h^2}{180}\left( 5f_{l,m,n} + 104f_{l+1,m,n} + 78f_{l+2,m,n} - 8f_{l+3,m,n} + f_{l+4,m,n}\right) \\ hu'_{l+3,m,n} - \frac{1}{2}u_{l+2,m,n} + \frac{1}{2}u_{l,m,n} &{}= \dfrac{h^2}{720}\left( 31f_{l,m,n} + 342f_{l+1,m,n} + 768f_{l+2,m,n} + 314f_{l+3,m,n} - 15f_{l+4,m,n}\right) \\ hu'_{l+4,m,n} - \frac{1}{2}u_{l+2,m,n} + \frac{1}{2}u_{l,m,n} &{}= \dfrac{h^2}{180}\left( 3f_{l,m,n} + 112f_{l+1,m,n} + 56f_{l+2,m,n} + 240f_{l+3,m,n} + 59f_{l+4,m,n}\right) \\ \end{aligned}$$

#### *Remark 1*

We note that the method (12) is locally obtained on $[x_l,x_{l+4}]$ and is applied to simultaneously obtain approximations to the semidiscretized system (3) over the whole 3D space $[L_1,L_2,L_3] \times [L_4,L_5,L_6];$ in which case $l=0(4)(L-4)$, $m=0(1)(M-1)$ and $n=0(1)(N-1)$. Also, we note that the first three formulas in (12) are of $O(h^8)$ while the derivative formulas are of $O(h^7)$.

### Convergence analysis

We discuss the convergence of the BVMs in the following theorem

#### **Theorem 2**

*Let*$\mathbf{U }$* be an approximation of the solution vector*${\overline{\mathbf{U }}}$* for the system obtained on a partition*$\pi _{L}:=\{L_1=x_{0}<x_{1}<\ldots <x_{L}=L_4,~ x_{m}=x_{m-1}+h\}$* from the method* (12).* If*$e_{l}=|u_{m,n}(x_{l})-u_{l,m,n}|$, $he^{\prime }_{l}=|hu^{\prime }_{m,n}(x_{l})-hu^{\prime }_{l,m,n}|$,* where the exact solution*$u_{m,n}(x)$* is several times differentiable on*$[L_1,L_4]$* and if*$\Vert E\Vert =\Vert \mathbf{U }-{\overline{\mathbf{U }}}\Vert$, *then, the BVM is convergent of order 6, which implies that *$\Vert E\Vert =O(h^{6})$.

#### *Proof*

We begin the proof by compactly writing (12) in matrix form with the introduction of the following matrix notations. Let A be a $2L\times 2L$ matrix defined by

$$\begin{aligned} A =\left[ \begin{array}{cc} A_{11} &{} A_{12} \\ A_{21} &{} A_{22} \end{array} \right] , \end{aligned}$$

$$\begin{aligned} A_{11}= \left[ \begin{array}{cccccc} 0 &{} -\frac{1}{2} &{} 0 &{} 0 &{} \cdots &{} 0\\ 1 &{} -\frac{1}{2} &{} 0 &{} 0 &{} \cdots &{} 0\\ 0 &{} -\frac{3}{2} &{} 1 &{} 0 &{} \cdots &{} 0\\ 0 &{} -2 &{} 0 &{} 1 &{} \cdots &{} 0\\ \vdots &{} &{} \ddots &{} &{} &{} \vdots \\ 0 &{} 0 &{} \cdots &{} -\frac{3}{2} &{} 1 &{} 0 \\ 0 &{} 0 &{} \cdots &{} -2 &{} 0 &{} 1 \\ \end{array} \right] , \end{aligned}$$

$$\begin{aligned} A_{21}= \left[ \begin{array}{cccccc} 0 &{} -\frac{1}{2} &{} 0 &{} &{} \cdots &{} 0\\ 0 &{} -\frac{1}{2} &{} 0 &{} &{} \cdots &{} 0\\ 0 &{} -\frac{1}{2} &{} 0 &{} &{} \cdots &{} 0\\ 0 &{} -\frac{1}{2} &{} 0 &{} &{} \cdots &{} 0\\ \vdots &{} &{} \ddots &{} &{} &{} \vdots \\ 0 &{} 0 &{} \cdots &{} &{} - \frac{1}{2} &{} 0 \\ \end{array} \right] , \end{aligned}$$

$A_{12}$ and $A_{22}$ are null and identity matrices respectively.

Similarly, let B be a $2L\times 2L$ matrix defined by

$$\begin{aligned} B =\left[ \begin{array}{cc} B_{11} &{} B_{12} \\ B_{21} &{} B_{22} \end{array} \right] , \end{aligned}$$

where $B_{ij}$ are given as

$$\begin{aligned} B_{11}= h^2 \left[ \begin{array}{cccccc} -\frac{144}{180} &{} -\frac{30}{180} &{} -\frac{16}{180} &{} \frac{3}{180} &{} 0 \cdots &{} 0\\ -\frac{204}{480} &{} -\frac{14}{480} &{} -\frac{4}{480} &{} \frac{4}{480} &{} 0 \cdots &{} 0\\ \frac{252}{480} &{} \frac{402}{480} &{} \frac{52}{480} &{} -\frac{3}{480} &{} 0 \cdots &{} 0\\ -\frac{16}{15} &{} -\frac{26}{15} &{} \frac{16}{15} &{} \frac{257}{15} &{} 0 \cdots &{} 0\\ &{} &{} \ddots &{} \ddots &{} &{} \\ 0 &{} \cdots 0&{} \frac{252}{480} &{} \frac{402}{480} &{} \frac{52}{480} &{} -\frac{3}{480}\\ 0 &{} \cdots 0&{} -\frac{16}{15} &{} -\frac{26}{15} &{} \frac{16}{15} &{} \frac{257}{15} \\ \end{array} \right] , \end{aligned}$$

$$\begin{aligned} B_{21}= h^2 \left[ \begin{array}{cccccc} \frac{70}{720} &{} -\frac{144}{720} &{} \frac{42}{720} &{} -\frac{7}{720} &{} 0 \cdots &{} 0\\ -\frac{104}{180} &{} \frac{78}{180} &{} -\frac{8}{180} &{} \frac{1}{180} &{} 0 \cdots &{} 0\\ \frac{342}{720} &{} \frac{768}{720} &{} \frac{314}{720} &{} -\frac{15}{720} &{} 0 \cdots &{} 0\\ \frac{112}{180} &{} -\frac{56}{180} &{} \frac{240}{180} &{} \frac{59}{180} &{} 0 \cdots &{} 0\\ &{} &{} \ddots &{} \ddots &{} &{} \\ 0 &{} \cdots 0&{} \frac{342}{720} &{} \frac{768}{720} &{} \frac{314}{720} &{} -\frac{15}{720}\\ 0 &{} \cdots 0&{} \frac{112}{180} &{} -\frac{56}{180} &{} \frac{240}{180} &{} \frac{59}{180} \\ \end{array} \right] , \end{aligned}$$

$B_{12}$ and $B_{22}$ are null matrices.

We also define the vectors

$$\begin{aligned} \mathbf{U } &{}= (u_{m,n}(x_1), \ldots , u_{m,n}(x_L),hu'_{m,n}(x_1), \ldots , hu'_{m,n}(x_L))^T\\ \mathbf{F } &{}= (f_{1,m,n}, \ldots , f_{L,m,n}, hf'_{1,m,n}, \ldots , hf'_{L,m,n})^T\\ \mathbf{C } &{}= \left( hu'_{0,m,n} + \frac{1}{2}u_{0,m,n} + \frac{53}{180}h^2f_{0,m,n}, - \frac{1}{2}u_{0,m,n} + \frac{19}{480}h^2f_{0,m,n},\right. \\ &\quad \frac{1}{2}u_{0,m,n} - \frac{17}{480}h^2f_{0,m,n}, -u_{0,m,n} - \frac{1}{15}h^2f_{0,m,n},0, \ldots ,0, \frac{1}{2}u_{0,m,n} - \frac{39}{720}h^2f_{0,m,n}, \\ &\quad\left. \frac{1}{2}u_{0,m,n} - \frac{5}{180}h^2f_{0,m,n}, \frac{1}{2}u_{0,m,n} - \frac{31}{720}h^2f_{0,m,n}, \frac{1}{2}u_{0,m,n} - \frac{3}{180}f_{0,m,n}, 0, \ldots , 0\right) ^T\\ L(h) &{}= (e_1, \ldots , e_L, he'_1, \ldots , he'_L)^T \end{aligned}$$

where *L*(*h*) is the local truncation error vector of the formulas in (12).

The exact form of the system formed by (12) is given by

13 $$\begin{aligned} A\mathbf{U } - B\mathbf{F }(\mathbf{U }) + \mathbf{C } + L(h) = 0, \end{aligned}$$

and the approximate form of the system is given by

14 $$\begin{aligned} A{\overline{\mathbf{U }}}- B\mathbf{F }({\overline{\mathbf{U }}}) + \mathbf{C } = 0, \end{aligned}$$

where ${\overline{\mathbf{U }}} = (u_{1,m,n}, \ldots , u_{L,m,n}, hu'_{1,m,n}, \ldots , hu'_{L,m,n})^T$ is the approximate solution of $\mathbf{U }$. Subtracting (13) from (14) and letting $\mathbf{E } = {\overline{\mathbf{U }}} - \mathbf{U } = (e_1, \ldots , e_L, he'_1, \ldots , he'_L)^T$ and using the Mean value theorem, we have the error system

15 $$\begin{aligned} (A - BJ)\mathbf{E } = L(h), \end{aligned}$$

where *J* is the Jacobian matrix and its entries $J_{11}$, $J_{12}$, $J_{21}$ and $J_{22}$ are defined as

$$\begin{aligned} J_{11} =\left[ \begin{array}{ccc} \frac{\partial f_1}{\partial y_1} &{} \ldots &{} \frac{\partial f_1}{\partial y_L}\\ \vdots &{} \vdots &{} \vdots \\ \frac{\partial f_L}{\partial y_1} &{} \ldots &{} \frac{\partial f_L}{\partial y_L}\\ \end{array} \right] , \end{aligned}$$

$$\begin{aligned} J_{12} =\left[ \begin{array}{ccc} \frac{\partial f_1}{\partial y'_1} &{} \ldots &{} \frac{\partial f_1}{\partial y'_L}\\ \vdots &{} \vdots &{} \vdots \\ \frac{\partial f_L}{\partial y'_1} &{} \ldots &{} \frac{\partial f_L}{\partial y'_L}\\ \end{array} \right] , \end{aligned}$$

$$\begin{aligned} J_{21} =h \left[ \begin{array}{ccc} \frac{\partial f'_1}{\partial y_1} &{} \ldots &{} \frac{\partial f'_1}{\partial y_L}\\ \vdots &{} \vdots &{} \vdots \\ \frac{\partial f'_L}{\partial y_1} &{} \ldots &{} \frac{\partial f'_L}{\partial y_L}\\ \end{array} \right] , \end{aligned}$$

$$\begin{aligned} J_{22} =h \left[ \begin{array}{ccc} \frac{\partial f'_1}{\partial y'_1} &{} \ldots &{} \frac{\partial f'_1}{\partial y'_L}\\ \vdots &{} \vdots &{} \vdots \\ \frac{\partial f'_L}{\partial y'_1} &{} \ldots &{} \frac{\partial f'_L}{\partial y'_L}\\ \end{array} \right] . \end{aligned}$$

Let $M = -BJ$ be a matrix of dimension 2*L* so that (15) becomes

16 $$\begin{aligned} (A + M)E = L(h), \end{aligned}$$

and for sufficiently small *h*, $A + M$ is a monotone matrix and thus nonsingular. Therefore

$$\begin{aligned} (A + M)^{-1} = D = (d_{ij}) \ge 0 \quad \text {and} \quad \sum _{j=1}^{2L}d_{ij} = O(h^{-2}), \end{aligned}$$

and

$$\begin{aligned} E &{}= DL(h),\\ ||E|| &{}= ||DL(h)||,\\ &{} =O(h^{-2})O(h^{8}),\\ &{}= O(h^{6}). \end{aligned}$$

which shows that the methods are convergent and the global error is of order $O(h^{6})$. $\square$

## Computational procedures

The method (12) can also be expressed in block form as

17 $$\begin{aligned} A_{0}\mathbf{V }_{\mu }= A_{1}\mathbf{V }_{\mu -1} + h^{2}B_{1} \mathbf{F }_{\mu -1}+h^{2} B_{0}\mathbf{F }_{\mu },~~\mu =1, \ldots ~~\Gamma , \end{aligned}$$

where the positive integer $\Gamma =N/4$ is the number of blocks,

$$\begin{aligned} \mathbf{V }_\mu &=(u_{l+1,m,n}, u_{l+2,m,n}, u_{l+3,m,n}, u_{l+4,m,n}, hu'_{l+1,m,n}, hu'_{l+2,m,n}, hu'_{l+3,m,n}, hu'_{l+4,m,n})^{T},\\ \mathbf{F }_\mu &=(f_{l+1,m,n}, f_{l+2,m,n}, f_{l+3,m,n}, f_{l+4,m,n}, hf'_{l+1,m,n},hf'_{l+2,m,n},hf'_{l+3,m,n}, hf'_{l+4,m,n})^{T}, \\ \mathbf{V }_\mu -1&=(u_{l-3,m,n}, u_{l-2,m,n}, u_{l-1,m,n}, u_{l,m,n}, hu'_{l-3,m,n}, hu'_{l-2,m,n}, hu'_{l-1,m,n}, hu'_{l,m,n})^{T},\\ \mathbf{F }_{\mu -1}&=(f_{l-3,m,n}, f_{l-2,m,n}, f_{l-1,m,n}, f_{l,m,n}, hf'_{l-3,m,n}, hf'_{l-2,m,n}, hf'_{l-1,m,n}, hf'_{l,m,n})^{T}, \end{aligned}$$

and $A_{0}$, $A_{1}$, $B_{0}$, and $B_{1}$ are matrices each of dimension 8 whose entries are given by the coefficients of (12).

Equation (1) is converted to (3) by discretizing the partition $\pi _M$ and $\pi _N$, given by

$$\begin{aligned} \pi _{M}&{}:=\{L_2=y_{0}<y_{1}<\cdots <y_{M}=L_5,~~~~~~ y_{m}=y_{m-1}+\Delta y\},\\ \pi _{N}&{}:=\{L_3=z_{0}<z_{1}<\cdots <z_{N}=L_6,~~~~~~ z_{n}=z_{n-1}+\Delta z\}, \end{aligned}$$

where

$$\begin{aligned} \Delta y = \dfrac{L_5-L_2}{M}, \quad \Delta x = \dfrac{L_6-L_3}{N} \end{aligned}$$

are constant stepsizes of the partition $\pi _M$ and $\pi _N$ respectively, $m=1(1)M$, $n=1(1)N,$*M* and *N* are positive integers and *m*, *n* are the grid index in the *y* and *z* direction respectively.

The resulting system of ODEs (3) is then solved on the partition $\pi _L$. The block unification of (17) lead to a large system of finite difference equations which is then solved to provide all solutions of (3) on $\Omega$.

The following algorithm summarizes the numerical integration of (3) for some set of points *x* on $\pi _L$.

*Step 1* Use the block unification of (17) for $\mu =1, n=0$ to obtain $\mathbf{V }_{1}$ on the domian $[x_{0}, L_2, L_3]\times [x_4, L_5, L_6]$ and for $\mu =2, n=4$, $\mathbf{V }_{2}$ is obtained on the domain $[x_{4}, L_2, L_3]\times [x_8, L_5, L_6]$, and on the domains $[x_{8}, L_2, L_3]\times [x_{12}, L_5, L_6], \ldots , [x_{L-4}, L_2, L_3]\times [x_L, L_5, L_6]$, for $\mu =3, \ldots ,\Gamma$, $n=8, 12 \ldots , N-4$, we obtain $\mathbf{V }_{3} \ldots , \mathbf{V }_{\Gamma }$.

*Step 2* The unified block given by the system $\mathbf{V }_{1} \bigcup \mathbf{V }_{2} \bigcup \ldots \bigcup \mathbf{V }_{\Gamma -1}\bigcup \mathbf{V }_{\Gamma }$ obtained in Step 1 is a large system with dimensions $2L(M-1)(N-1)$ with $u_{m,n}(x_l) \approx u(x_l, y_m, z_n)$, $l=1, \ldots , L$, $m=1, \ldots , M$, $n=1, \ldots , N.$

*Step 3* The system obtained in Step 2 is solved using the feature NSolve in Mathematica 10.0 for linear problems and FindRoot (which incorporates the Newton’s method) for nonlinear problems.

*Step 4* The solution of (1) is approximated by the solution in Step 3 as $$\mathbf{U }(x_l) = [u(x_l, y_m, z_n), \ldots , u(x_L, y_m, z_n)]^T$$, $$m=1,\ldots , M$$$$n=1, \ldots , N.$$

## Numerical examples

In this section, some examples are investigated to show the reliability and efficiency of the proposed scheme in this paper.

### Test 1

**Fig. 1:**
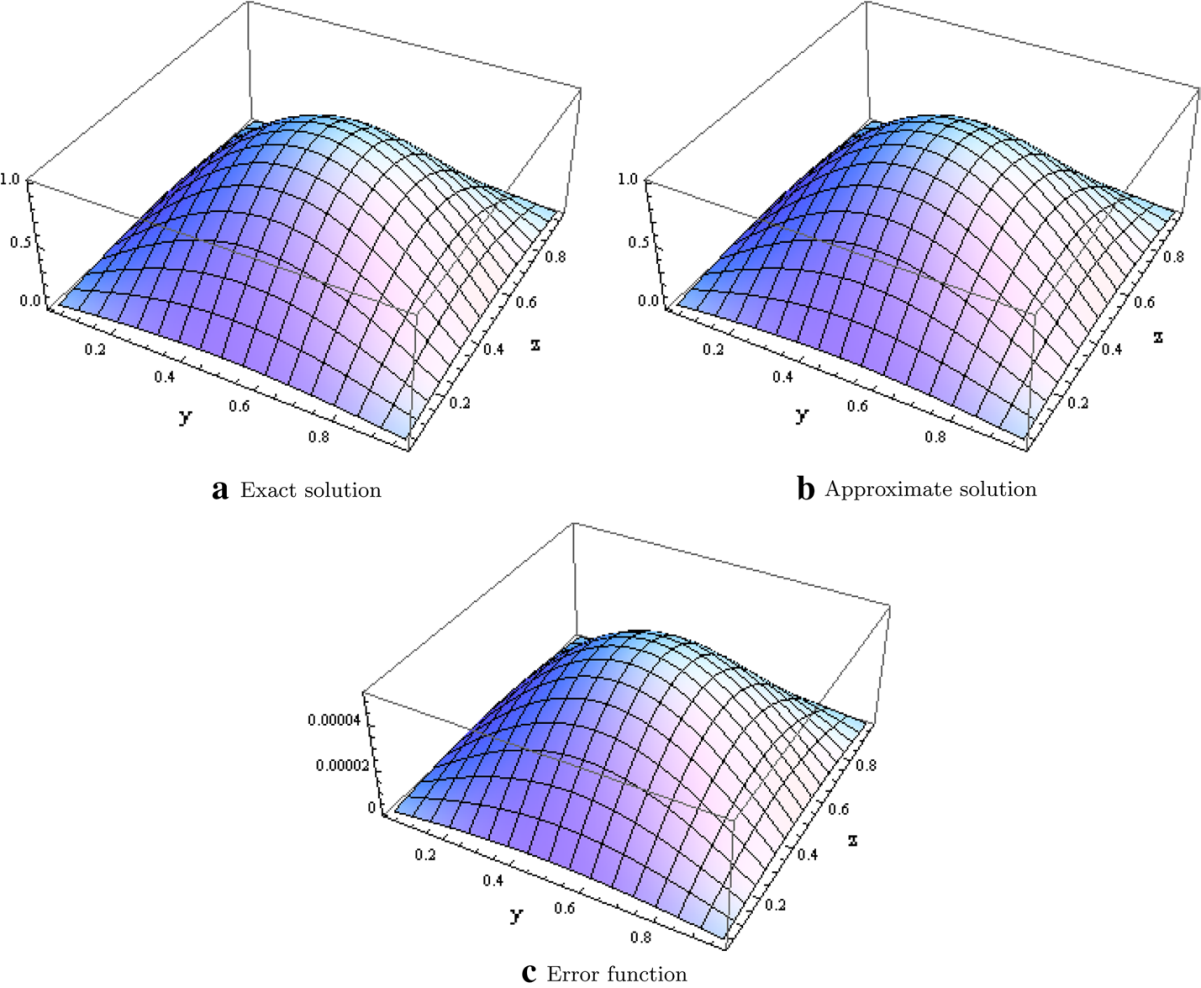
Graphical evidence when $x=0.5$ and $L_4 = 1.0$ for test problem 1

We consider the Laplace equation with non zero forcing term *G*(*x*, *y*, *z*) and with zero boundary values on the entire $\partial \Omega$ given in Zhang (1998c) and whose solution is given as

$$\begin{aligned} u(x,y,z) &{}= \sin (\pi x) \sin (\pi y) \sin (\pi z)\\ G(x,y,z) &{}=-3\pi ^2 \sin (\pi x) \sin (\pi y) \sin (\pi z) \end{aligned}$$

where $\Omega = [0,1]^2 \times [0,L_4]$. Table 1 shows the errors in the $l^\infty$ norm with different mesh sizes. Figure 1 shows the plot of the exact, approximate and error function when $x=0.5.$

**Table 1:** Errors in the $l^\infty$ norm for test problem 1

$L_4$	$\Delta y = \Delta z =0.25$	$\Delta y = \Delta z =0.125$	$\Delta y = \Delta z =0.0625$	$\Delta y = \Delta z =0.03125$
0.1	1.903e−04	4.905e−05	1.242e−05	3.109e−06
0.2	1.413e−03	3.641e−04	9.208e−05	2.305e−05
0.3	4.238e−03	1.091e−03	2.754e−04	6.896e−05
0.4	8.610e−03	2.213e−03	5.576e−04	1.396e−04
0.5	1.401e−02	3.586e−03	9.028e−04	2.260e−04
0.6	1.972e−02	5.010e−03	1.263e−03	3.158e−04
0.7	2.506e−02	6.291e−03	1.592e−03	3.980e−04
0.8	2.953e−02	7.340e−03	1.855e−03	4.636e−04
0.9	3.280e−02	8.142e−03	2.029e−03	5.092e−04
1.0	3.472e−02	8.616e−03	2.144e−03	5.356e−04

### Test 2

**Fig. 2:**
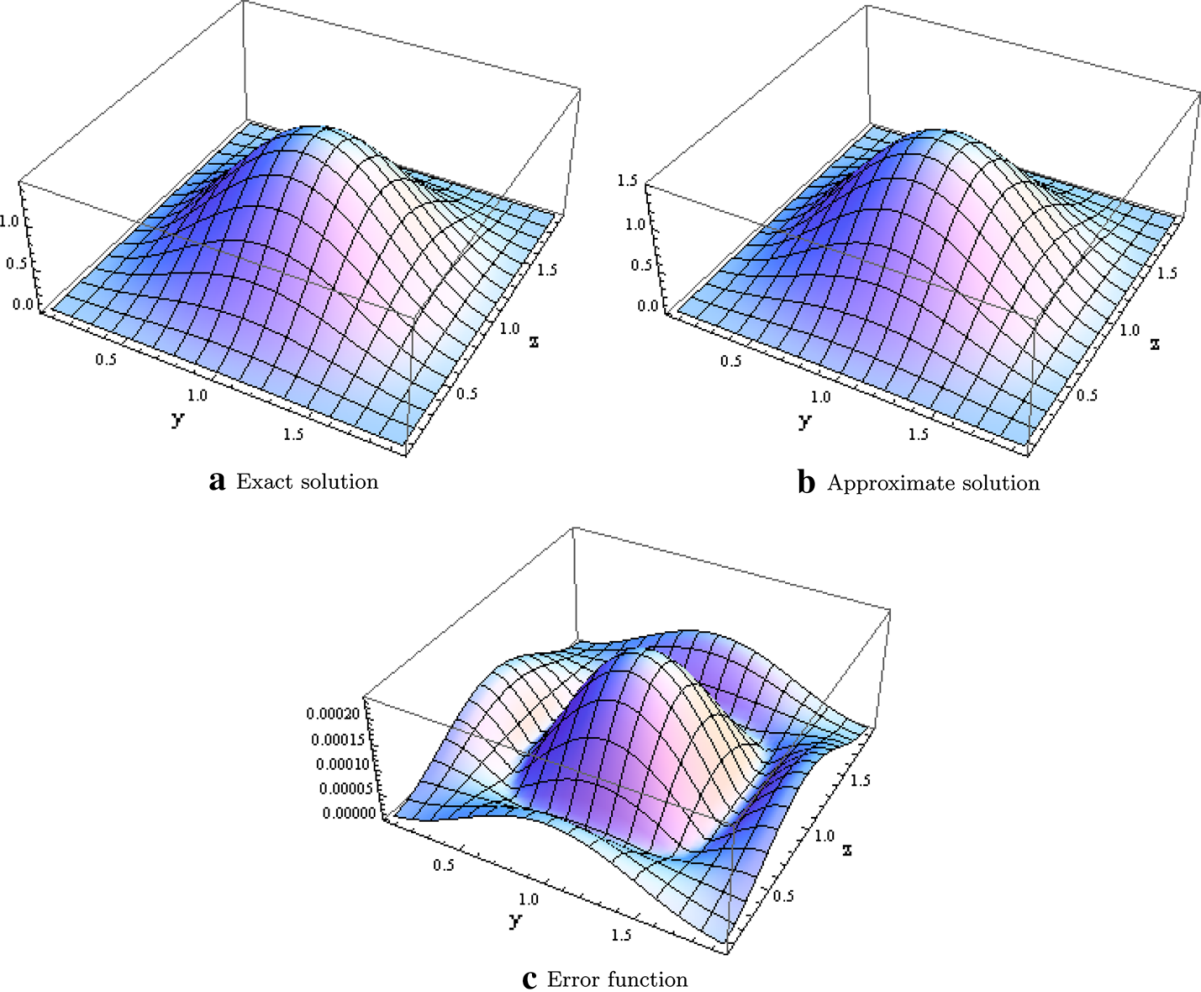
Graphical evidence when $x=1.0$ and $L_4 = 2.0$ for test problem 2

Next, we consider the following Sine-Gordon equation given in Cui (2010)

$$\begin{aligned} \begin{aligned} &\dfrac{\partial ^2u}{\partial x^2}+ \rho \dfrac{\partial u}{\partial x} = \dfrac{\partial ^2u}{\partial y^2} + \dfrac{\partial ^2u}{\partial z^2} -2\sin (u) + 2\sin (e^{-\alpha x}(1 - \cos (\pi y))(1 - \cos (\pi z) ))\\&\quad- e^{-\alpha x}\left[ \alpha (\rho - \alpha )(1 - \cos (\pi y))(1 - \cos (\pi z))\right] \\&\quad+ \pi ^2 (\cos (\pi y) + \cos (\pi z) - 2\cos (\pi y)\cos (\pi z)), \qquad 0<y,z<2, t>0\\ &u(0,y,z)= (1 - \cos (\pi y))(1 - \cos (\pi z)), \quad 0<y,z<2, \\ &\dfrac{\partial u}{\partial x}(x,y,z)| _{x=0}= -\alpha (1 - \cos (\pi y))(1 - \cos (\pi z)), \quad 0<y,z<2, \\ &u(x,0,z)= u(x,2,z) = u(x,y,0) = u(x,y,2) = 0, \quad t>0. \end{aligned} \end{aligned}$$

where $\Omega = [0,2]^2 \times [0,L_4]$ and whose theoretical solution is $u(x,y,z) = e^{-\alpha x}(1 - \cos (\pi y))(1 - \cos (\pi z))$. In our computations, we have chosen $\alpha = \rho = 1$ and the $l^\infty$ norms are given in Table 2 with different meshsizes. Figure 2 also shows the plot of the exact, approximate and error function when $x=1.$

**Table 2:** Errors in the $l^\infty$ norm for test problem 2

$L_4$	$\Delta y = \Delta z =0.5$	$\Delta y = \Delta z =0.25$	$\Delta y = \Delta z =0.125$	$\Delta y = \Delta z =0.0625$
0.2	1.266e−01	3.343e−02	8.471e−03	2.125e−03
0.4	4.016e−01	1.035e−02	2.609e−02	26.535e−03
0.6	6.692e−01	1.637e−02	4.067e−02	1.015e−02
0.8	8.183e−01	1.811e−01	4.406e−02	1.112e−02
1.0	8.115e−01	1.815e−01	4.406e−02	1.013e−02
1.2	8.344e−01	1.814e−01	4.406e−02	1.015e−02
1.4	7.892e−01	1.788e−01	4.380e−02	1.187e−02
1.6	8.224e−01	1.810e−01	4.380e−02	1.055e−02
1.8	8.429e−01	1.764e−01	4.371e−02	1.034e−02
2.0	8.308e−01	1.815e−01	4.408e−02	1.154e−02

### Test 3

**Fig. 3:**
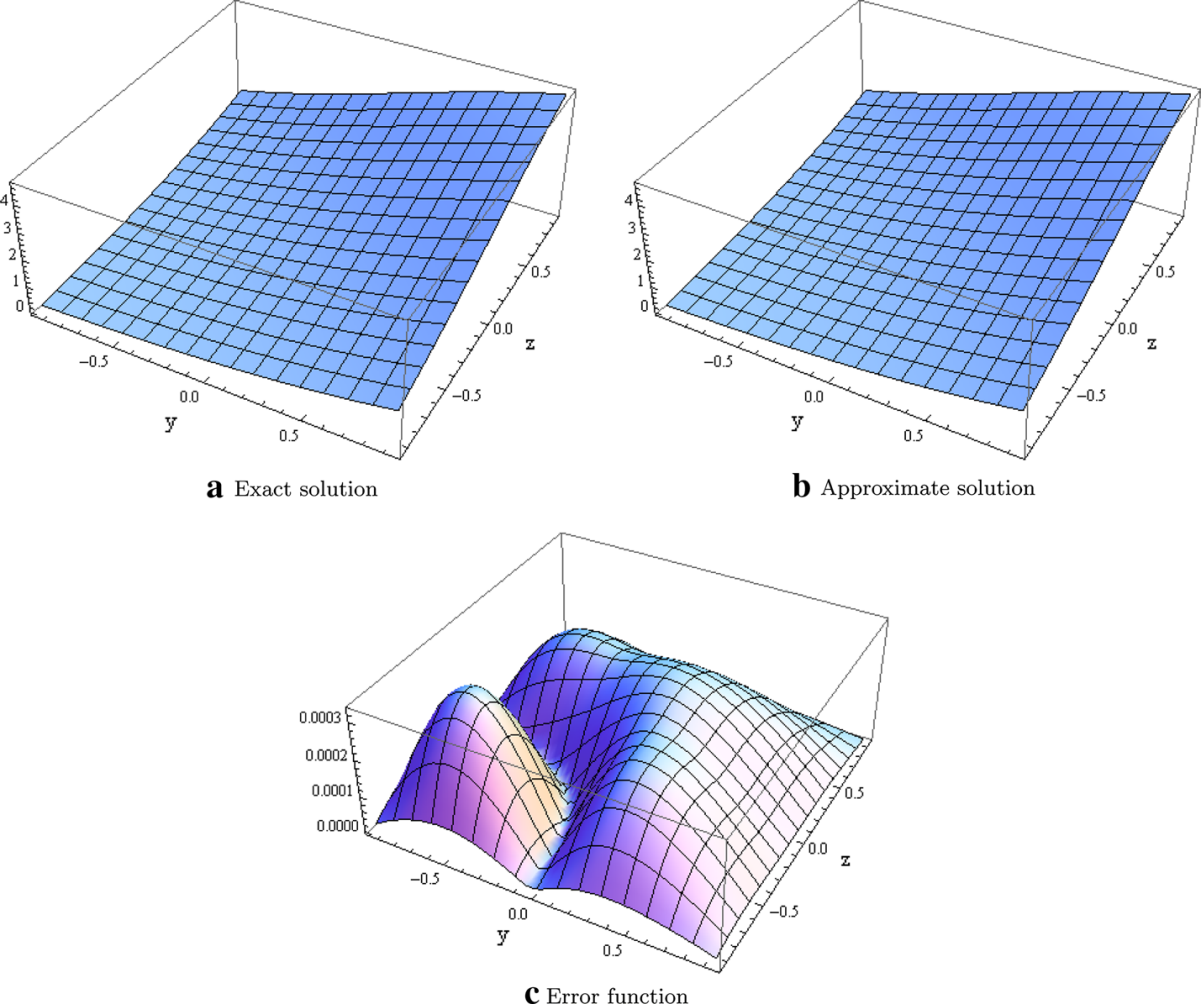
Graphical evidence when $x=1.0$ and $L_4 = 1.0$ for test problem 3

We also consider the following equation given in Cui (2010)

$$\begin{aligned} \begin{aligned} &\dfrac{\partial ^2u}{\partial x^2}= \dfrac{\partial ^2u}{\partial y^2} + \dfrac{\partial ^2u}{\partial z^2} - \sin (u) \\ &u(0,y,z)= 4\arctan (e^{y+z}), \quad -1<y,z<1, \\ &\dfrac{\partial u}{\partial x}(x,y,z)| _{x=0}= \dfrac{4e^{y+z}}{1 + e^{2y+2z}}, \quad -1<y,z<1, \\ \end{aligned} \end{aligned}$$

where $\Omega = [-1,1]^2 \times [-1,L_4]$ and whose theoretical solution is $u(x,y,z) = 4\arctan (e^{y+z-x})$ with corresponding Dirichlet boundary conditions. The $l^\infty$ norms are given in Table 3 with different meshsizes. Figure 3 also shows the plot of the exact, approximate and error function when $x=1.0.$

**Table 3:** Errors in the $l^\infty$ norm for test problem 3

$L_4$	$\Delta y = \Delta z =0.5$	$\Delta y = \Delta z =0.25$	$\Delta y = \Delta z =0.125$	$\Delta y = \Delta z =0.0625$
$-0.8$	5.340e−04	1.493e−04	3.896e−05	9.775e−06
$-0.6$	2.042e−03	5.911e−04	1.493e−03	3.763e−05
$-0.4$	4.878e−03	1.257e−03	3.167e−04	7.991e−05
$-0.2$	7.896e−03	2.007e−03	5.273e−04	1.320e−05
0.0	1.017e−02	3.005e−03	7.539e−04	1.895e−04
0.2	1.502e−02	3.966e−03	9.964e−04	2.494e−04
0.4	1.739e−02	4.742e−03	1.231e−03	3.097e−04
0.6	2.005e−02	5.389e−03	1.436e−03	3.648e−04
0.8	2.324e−02	5.975e−03	1.514e−03	3.793e−04
1.0	1.995e−02	5.048e−03	1.349e−03	3.449e−04

### Test 4

**Fig. 4:**
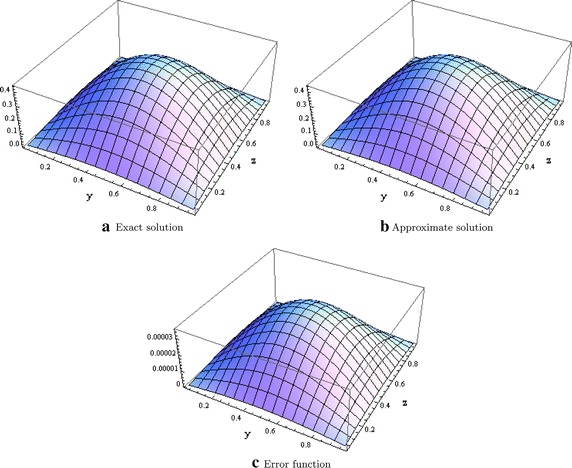
Graphical evidence when $x=0.5$ for test problem 4

We also consider the singularly perturbed convection diffusion equation given in Mohanty and Singh (2006)

$$\begin{aligned} \epsilon \left( \dfrac{\partial ^2u}{\partial x^2} + \dfrac{\partial ^2u}{\partial y^2} + \dfrac{\partial ^2u}{\partial z^2}\right) = \dfrac{\partial u}{\partial x} \end{aligned}$$

defined in the domain $\Omega = [0,1]^3$ with boundary $\partial \Omega$ and subject to the Dirichlet boundary conditions and whose theoretical solution is

$$\begin{aligned} u(x,y,z) = e^{\frac{x}{2\epsilon }}\dfrac{\sin (\pi y)\sin (\pi z)}{\sinh (\sigma )}\left[ 2e^{-\frac{1}{2\epsilon }}\sinh (\sigma x) + \sinh (\sigma (1-x))\right] . \end{aligned}$$

where $\sigma ^2 = 2\pi ^2 + \dfrac{1}{4\epsilon ^2}$. We have solved the problem using $\epsilon =0.1$ to 1.0. The $l^\infty$ norms are given in Table 4 with different meshsizes. Figure 4 also shows the plot of the exact, approximate and error function when $x=0.5.$

**Table 4:** Errors in the $l^\infty$ norm for test problem 4

$\epsilon$	$\Delta y = \Delta z =0.25$	$\Delta y = \Delta z =0.125$	$\Delta y = \Delta z =0.0625$	$\Delta y = \Delta z =0.03125$
0.1	1.492e−01	2.086e−02	1.801e−03	2.767e−04
0.2	2.830e−02	5.918e−03	1.108e−03	2.626e−04
0.34	1.890e−2	4.807e−03	1.113e−03	2.742e−04
0.4	1.655e−02	4.744e−03	1.146e−03	2.843e−04
0.5	1.680e−02	4.805e−03	1.174e−03	2.916e−04
0.6	1.737e−02	4.867e−03	1.195e−03	2.916e−04
0.7	1.780e−02	4.919e−03	1.211e−03	3.970e−04
0.8	1.813e−02	4.962e−03	1.224e−03	3.048e−04
0.9	1.839e−02	4.998e−03	1.235e−03	3.075e−04
1.0	1.860e−02	5.028e−03	1.243e−03	3.097e−04

### Test 5

**Fig. 5:**
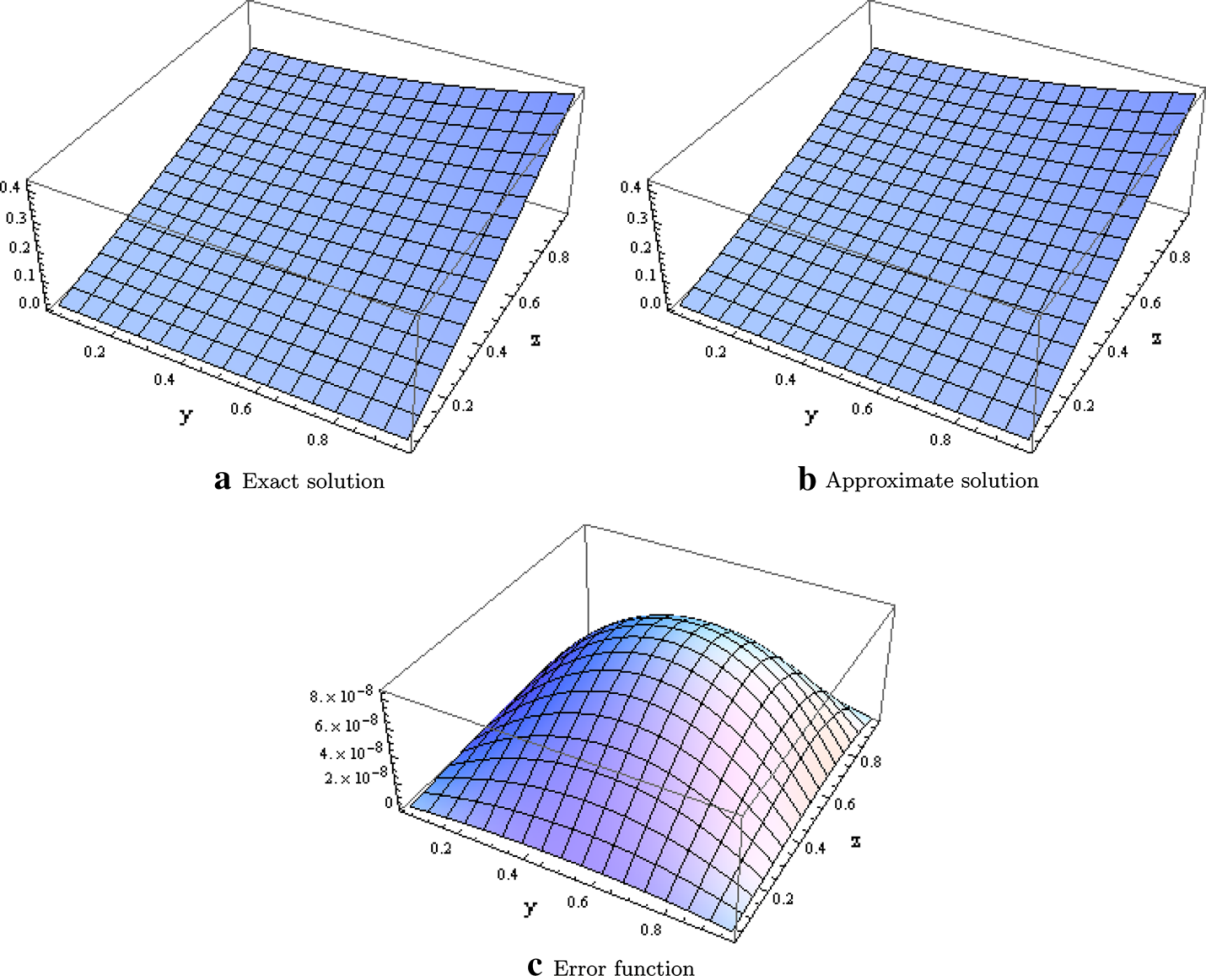
Graphical evidence when $x=0.5$ for $\alpha =1$ for test problem 5

The singularly perturbed elliptic boundary value problem given in Mohanty and Singh (2006)

$$\begin{aligned} \epsilon \left( \dfrac{\partial ^2u}{\partial x^2} + \dfrac{\partial ^2u}{\partial y^2} + \dfrac{\partial ^2u}{\partial z^2} +\dfrac{\alpha }{x}\dfrac{\partial u}{\partial x}\right) = G(x,y,z) \end{aligned}$$

defined in the domain $\Omega = [0,1]^3$ with boundary $\partial \Omega$ and subject to the Dirichlet boundary conditions and where the forcing term G is set to satisfy the exact solution given as $u(x,y,z) = x^2\cosh (y)\sinh (z)$. We have solved the problem using $\epsilon =0.001$. The $l^\infty$ norms are given in Table 5 with different meshsizes and for different values of $\alpha$. Figure 5 also shows the plot of the exact, approximate and error function when $x=0.5.$

**Table 5:** Errors in the $l^\infty$ norm for different values of $\alpha$ for test problem 5

$\alpha$	$\Delta y = \Delta z =0.25$	$\Delta y = \Delta z =0.125$	$\Delta y = \Delta z =0.0625$	$\Delta y = \Delta z =0.03125$
0	1.359e−04	3.664e−05	9.521e−06	2.395e−06
1	1.383e−04	3.725e−05	9.768e−05	3.226e−06
2	1.380e−04	3.951e−05	9.858e−05	1.892e−06
3	1.370e−04	4.582e−05	1.649e−05	8.940e−05
4	1.361e−04	5.382e−05	2.623e−05	1.892e−05
5	1.359e−04	6.466e−05	3.936e−05	3.226e−05

## Conclusion

In this paper, we have developed a highly accurate 3D problem solver. This has been achieved by the discretization of two of the spatial variables and the construction of a continuous BVM via the interpolation and collocation approach for solving the resulting semidiscretized system. The results given in "Numerical examples" show that the approach is highly efficient and highly accurate.
